# The BtaF Trimeric Autotransporter of *Brucella suis* Is Involved in Attachment to Various Surfaces, Resistance to Serum and Virulence

**DOI:** 10.1371/journal.pone.0079770

**Published:** 2013-11-13

**Authors:** Verónica Ruiz-Ranwez, Diana M. Posadas, Silvia M. Estein, Patricia L. Abdian, Fernando A. Martin, Angeles Zorreguieta

**Affiliations:** 1 Fundación Instituto Leloir and IIBBA CONICET, Buenos Aires, Argentina; 2 Departamento de Química Biológica, Facultad de Ciencias Exactas y Naturales, Universidad de Buenos Aires, Buenos Aires, Argentina; 3 Laboratorio de Inmunología, Facultad de Ciencias Veterinarias, Centro de Investigación Veterinaria de Tandil (CIVETAN), Universidad Nacional del Centro de la Provincia de Buenos Aires, Tandil, Provincia de Buenos Aires, Argentina; East Carolina University School of Medicine, United States of America

## Abstract

The adhesion of bacterial pathogens to host cells is an event that determines infection, and ultimately invasion and intracellular multiplication. Several evidences have recently shown that this rule is also truth for the intracellular pathogen *Brucella*. *Brucella suis* displays the unipolar BmaC and BtaE adhesins, which belong to the monomeric and trimeric autotransporter (TA) families, respectively. It was previously shown that these adhesins are involved in bacterial adhesion to host cells and components of the extracellular matrix (ECM). In this work we describe the role of a new member of the TA family of *B. suis* (named BtaF) in the adhesive properties of the bacterial surface. BtaF conferred the bacteria that carried it a promiscuous adhesiveness to various ECM components and the ability to attach to an abiotic surface. Furthermore, BtaF was found to participate in bacterial adhesion to epithelial cells and was required for full virulence in mice. Similar to BmaC and BtaE, the BtaF adhesin was expressed in a small subpopulation of bacteria, and in all cases, it was detected at the new pole generated after cell division. Interestingly, BtaF was also implicated in the resistance of *B. suis* to porcine serum. Our findings emphasize the impact of TAs in the *Brucella* lifecycle.

## Introduction

Species from the *Brucella* genus are Gram negative bacteria, facultative intracellular pathogens responsible for brucellosis, a zoonotic disease that affects several terrestrial and marine mammals, including livestock [1]. *Brucella abortus*, *Brucella melitensis*, and *B. suis* are the most economically significant species of the group, since their preferred hosts are cattle, caprine, and swine, respectively. Brucellosis causes abortion and infertility in animals. In humans it may lead to a severe debilitating disease [2]. Human brucellosis is acquired either through consumption of contaminated dairy products or by coming into contact with infected animal secretions [3], [4].

When brucellae are ingested or inhaled, they cross the mucosal surfaces and are transported to the lymph nodes by phagocytic cells. The spread and multiplication in lymph nodes, liver, spleen, bone marrow and other tissues occur via macrophages [5], [6]. During the onset of the infection, *Brucella* is able to resist the killing action of several bactericidal substances, including the complement present in serum [7], [8]. It was proposed that *Brucella* lipopolysaccharide hampers the binding of complement activating components to the bacterial surface [9]. It is accepted that a low activation of the innate immunity for a long incubation period opens an immunological window that gives the opportunity to brucellae to spread throughout the reticuloendothelial system and establish the intracellular replication niche. After this long incubation period, a strong adaptive immunity is induced and the clinical symptoms are evident [5]. The virulence of *Brucella* spp. depends on their ability to replicate and survive within macrophages and other host cells, including epithelial cells, in a compartment derived from the endoplasmic reticulum [10], [11], [12], [13].

Intracellular pathogens must bind to the cells or other host components to successfully infect the host. Bacteria use a great variety of tools to adhere and eventually invade the host cell, ranging from multimeric complexes, such as pili or fimbria, to monomeric or oligomeric proteins. Bacterial adhesins mediate the initial interaction with the host by recognition of different host molecules, including components of the extracellular matrix (ECM), integrins or integral host membrane proteins [14]. *B. abortus* and *B. melitensis* bind to HeLa and macrophages cells with a characteristic kinetics. It was suggested that this process is mediated by host cell molecules rich in sialic acid residues through the *Brucella* surface UgpB protein [15], [16]. *Brucella* also interacts with components of the ECM such as fibronectin [15]. We have identified by phage display a large fibronectin-binding protein of 340 kDa (BmaC) from *B. suis*, which belongs to the monomeric (type I) autotransporter family. Members of this family are extracellular proteins that contain a C-terminal β-barrel domain responsible for the translocation of the functional domain across the outer membrane [17], [18]. We demonstrated that BmaC is involved in the binding of *B. suis* to non-phagocytic cells, such as HeLa and A549 epithelial cells, through interaction with cellular fibronectin [19]. We have recently identified BtaE, a member of the type II (trimeric) autotransporter family, which was shown to contribute to the adhesion of *B. suis* to hyaluronic acid and epithelial cells, and was necessary for full virulence in mice [20]. Members of the trimeric autotransporter (TA) family also have the information in the C-terminal region for their own translocation through the outer membrane but to do so they form trimeric structures on the bacterial surface [17]. Remarkably, we found that both, the BmaC and BtaE adhesins are associated exclusively with the new cell pole, suggesting that this pole in *Brucella* is specialized for adhesion [20]. Besides, these findings support the concept that bacterial polarity is an important feature of *Brucella* physiology [21], [22], [23].

*In silico* analysis led to the identification of a second protein of the TA family, which we named BtaF. Through heterologous expression and mutational approaches we show that BtaF plays a role in the adhesive features of the *Brucella* cell surface. Our observations suggest that some of BtaE and BtaF functions partially overlap while others are distinctive of BtaF. In fact, BtaF (but not BtaE) confers resistance to complement activity. Interestingly, both adhesins are required to achieve full infectivity in the mouse model.

## Materials and Methods

### Ethical statement

Animal procedures and management protocols were approved by the Animal Welfare and Ethics Committee according to Animal Welfare Policy (Act 087/02) of the Faculty of Veterinary Medicine (Universidad Nacional del Centro de la Provincia de Buenos Aires, Tandil, Argentina; http://www.vet.unicen.edu.ar).

### Bacterial strains, cell culture and media

*E. coli* strains used in this study (DH5α, K12 and derivatives) were grown at 37°C in Luria Bertani (LB) medium. Antibiotics were added when needed: ampicillin (200 µg/ml), chloramphenicol (50 µg/ml), and tetracycline (5 µg/ml). *B. suis* M1330 [ATCC 23444] and derivative strains were grown at 37°C in Bacto Tryptic Soy Broth (TSB, Bacto). When necessary antibiotics were added: chloramphenicol (6 µg/ml), kanamycin (50 µg/ml) and nalidixic acid (10 µg/ml). HeLa cells and lung epithelial A549 (ATCC CCL 185) cells were cultured in DMEM (GIBCO), and murine J774 macrophages in RPMI (GIBCO) media; both supplemented with 5% foetal calf serum (PAA), at 37°C in a 5% CO_2_ atmosphere.

### Molecular techniques

All DNA manipulations were carried out by using standard procedures. Sequencing was done by cycle sequencing performed with a BigDye kit (Applied Biosystems) in a 3130 Genetic Analyzer (Applied Biosystems). Sequence data of *B. suis* genome [24] was obtained from the *Brucella* Bioinformatical Portal (BBP) website (http://www.phidias.us/bbp/data/). PCR primers containing added restriction enzymes were designed to amplify the flanking regions of *btaF*. Primers F1 (5′ GGGCATGCTGTTCTGGACAGGCGTAGCTG 3′) and R1 (5′ CGCTGCAGCTCCGCCTTCGCAGCGTCG 3′) amplified a region of 371 pb upstream *btaF*; and primers F2 (5′ GCCTGCAGCATGGCATAATATGCACCCG 3′) and R2 (5′ CGGGATCCACCCAGACATCGCCGCCGC 3′) a region of 379 pb downstream the gene. New restriction sites are underlined. The generated PCR fragments were purified, cleaved with the corresponding enzymes, ligated together, and cloned into the pK18mobsacB mobilizable suicide vector (Km resistant) [25] obtaining pKmobsacΔ*btaF* plasmid. This plasmid was conjugated into a nalidixic resistant-derivative of the wild type strain *B. suis* M1330 and double recombinant clones were selected (Nal^R^, Km^S^, sucrose resistant). The clean deletion of the entire open reading frame was confirmed by PCR, and the absence of BtaF was confirmed by Western blot. The *btaF* gene (including its own promoter) was cloned into the pBBR1MCS-1 broad host range cloning plasmid [26]. A region of 1645 pb containing 631 pb of the upstream region, the *btaF* ORF and 177 pb corresponding to the downstream region was amplified using the following primers: FComp (5′ CTGGTACCAGCGCTGGTGCAGAAGGTGATAGAC 3′) and RComp (5′ CTGAGCTCCAATGCCCGCATGTGACGTTGCAAT 3′). New restriction sites are underlined. The amplified product was cloned into the pBBR1MCS-1 vector [27] resulting in the pBBR*btaF* plasmid, which was conjugated into *B. suis* Δ*btaF*. Transconjugants were selected (Cm^R^) and confirmed by PCR. *E. coli* K12 was transformed with pBBR*btaF* or empty pBBR1MCS-1 vector as control. Transformed clones were selected (Cm^R^) and confirmed by PCR.

### In silico analysis

Protein domains were analysed with Pfam [28], BLAST [29], and daTAA [30], which is specific for trimeric autotransporters. Secretion signal was identified with SignalP Server [31]. Alignments of the proteins were made with ClustalX (1.81) [32].

### Binding to host components

The affinity of BtaF-expressing bacteria to ECM components and fetuin (as a compound rich in sialic acid) was performed as described elsewhere [33]. Briefly, 96-well plates (Nunc Maxisorp) were coated overnight at 4°C with 50 µl of 100 µg/ml solutions of the ligand and dissolved in phosphate buffered saline (PBS). Bacteria were grown overnight, washed, and resuspended in PBS to a final concentration of 1×10^9^ colony forming units (CFU)/ml (OD = 1 for *E. coli* and OD = 0.2 for *Brucella*). Wells were washed three times with 100 µl of PBS to eliminate unbound ligand. Then, 50 µl of bacterial suspensions were added to each well and incubated at 37°C for 3 hours. After incubation, wells were washed three times with PBS to remove non-adherent bacteria, and adherent bacteria were harvested with trypsin-EDTA (0.05% trypsin (Gibco®) −0.5% EDTA (USB). After incubating for 10 minutes at 37°C, serial dilutions were done, plated on LB or TSB agar with the appropriate antibiotic, and CFU were determined.

### Attachment to abiotic surfaces

Bacterial attachment to wells of polystyrene (PE) plates was assayed based on previous protocol [34]. Briefly, starter cultures were grown in appropriate media overnight and diluted on fresh media (1∶1000); then 150 µl of these diluted cultures were seeded in the wells of PE 96-well flat bottom culture plates (Cellstar, Greiner Bio One). These plates were then cultured at 37°C for 24 hours with agitation. Unbound bacteria were removed by gently washing the wells three times with NaCl 0.9%, and attached bacteria were quantified by staining with 0.1% (w/v in water) crystal violet (CV) (Acros Organics, Geel, Belgium). Optical density was measured at λ = 595 nm in a Microplate Reader (DTX 880 multimode detector, Beckman Coulter).

### Autoagregation

Bacteria were cultured overnight on appropriate media at 37°C with agitation. Then a dilution was carried on (1∶100) and cells were grown overnight. The cultures were left at room temperature without agitation. At different time points the percentage of the tube that had bacteria in suspension was measured.

### Antibodies

**Figure 1:**
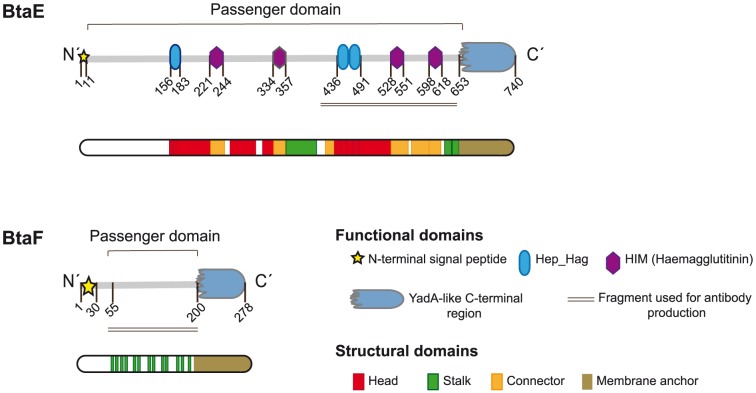
BtaE and BtaF domain organization. Schematic representation of BtaE and BtaF showing functional and structural domains predicted by bioinformatics (SignalP, Pfam, BLAST and daTAA), and the fragment of the protein used for antibody production (underlined region). Numbers indicate amino acid positions within the adhesin.

Polyclonal antiserum was obtained by immunization of mice with a peptide corresponding to BtaF passenger domain (Fig. 1, underlined region). The coding sequence was amplified using the primers FAb (5′CCCATATGCTAATTAGTGAAAATAGGCAAG 3′) and RAb (5′CAGTCGACTCCGGCACGTGCCTC 3′), and cloned into pET28a (Novagen). New restriction sites are underlined. The recombinant peptide was purified by HPLC and then separated by sodium dodecyl sulphate (SDS)-PAGE from other proteins. The band corresponding to the peptide was cut from the gel and then lyophilized. The powder was resuspended in sterile PBS and then used for immunization of five C57/BL6 male mice. Animal procedures were performed in compliance with institutional, as well as governmental, rules and regulations.

### Western blot

Total proteins from both *Brucella* and *E. coli* strains were separated on a semi-native gel, based on a protocol previously described [35]. Proteins from the extracellular medium of *Brucella* were also analysed; 50 ml of cultured bacteria were centrifuged to separate supernatant (containing secreted compounds) from bacterial cells. Extracellular proteins were precipitated with 10% trichloroacetic acid, and resuspended in sample buffer (see below). The cell pellet fraction was resuspended in cold 20 mM Tris-HCl buffer (pH 8.0), 1 mM phenylmethylsulfonyl fluoride (PMSF) and disrupted using Precellys (Bertin technologies). This homogenate was kept on ice (or at −20°C for storage). Then one volume (1∶1) of sample buffer (20 mM Tris pH 8, 2 mM EDTA pH 8, 40% sucrose, 0.02% bromo phenol blue, 0.05% SDS for *E. coli* or 0.1% for *B. suis*) was added and incubated for few minutes on ice. Next, samples were subjected to 10% polyacrylamide gel electrophoresis, which was carried out at 4°C to prevent protein denaturation. Resolved proteins were transferred to PDVF membranes (GE Healthcare), blocked overnight with TBS 5% milk powder (w/v) at 4°C with gentle agitation. Blots were probed with the polyclonal mice anti-BtaF serum (1∶300 for *E. coli*, and 1∶800 for *Brucella*) and a goat HRP-conjugated secondary anti-mouse antibody (1∶30.000, Santa Cruz). Finally, blots were revealed using ECL Plus (Amersham). BtaF antiserum was pre-adsorbed with an *E. coli* extract carrying the empty pET28a vector or *B.suis* Δ*btaE*Δ*btaF*.

### Immunofluorescence

*Brucella* was transformed with a derivative of pBBR1MCS-2 [26], [27], which carries *gfp* constitutively expressed (excitation at 488 nm, emission at 509 nm, filters used BP 505–530) [19]. The strains carrying polar markers fused to fluorescent proteins were obtained as previously described by biparental conjugation with *E. coli* S17.1 strains carrying either *pdhS-egfp* [21] or *aidB-yfp* [36]. *E. coli* strains were transformed with the plasmid pHC60 [37] which carries *gfp c*onstitutively expressed. BtaF was detected by immnufluorescence using anti-BtaF antibodies (see above) as previously described [20].

### Cell infection assays

Infection assays were carried out as previously described [20]. Briefly, HeLa (ATCC CCL2, human epithelial carcinoma cell line), A549 (ATCC CCL185, human lung epithelial cells) or murine macrophages (J774) cell lines were seeded in 24-well plates and inoculated with the different strains (multiplicity of infection 100∶1). Total bacteria associated with the cells were determined by lysis with 0.1% Triton X-100, after 1 hour or 45 minutes (in the case of macrophages) of incubation and plating serial dilutions. To quantify the number of intracellular viable bacteria a standard gentamicin protection assay was performed. The number of adherent bacteria was calculated as the difference between total bacteria associated to the cells and intracellular bacteria. A similar procedure was performed to evaluate adhesion of recombinant *E. coli* to HeLa cells.

### Virulence in BALB/c mice

Six to eight weeks-old female BALB/c mice were purchased from the animal facility from Leloir Institute. They were randomly distributed in experimental groups at least 1 week before being inoculated. The animals were housed in a filter-ventilated containment in the Laboratory Animal Facility of the Facultad de Ciencias Veterinarias (Tandil) receiving water and food *ad libitum*. All experimental protocols were performed by the premise of minimizing the suffering to which animals are exposed and using the minimum number of experimental animals to ensure statistically significant results. BALB/c mice (10 per group) were inoculated by intragastric delivery of wild type *B. suis* 1330 (1.35×10^8^ CFU/mouse), and its isogenic derivative strains Δ*btaE* (1.29×10^8^ CFU/mouse), Δ*btaF* (1.34×10^8^ CFU/mouse), Δ*btaF* pBBR*btaF* (1.27×10^8^ CFU/mouse) and Δ*btaE*Δ*btaF* (1.48×10^8^ CFU/mouse), suspended in 300 µl of 10% sodium bicarbonate by use of a plastic feeding tube introduced through the mouth. Five mice from each group were sacrificed at 7 and 30 days postinfection (p.i.), and spleens were removed. Dilutions of spleen homogenates were plated in duplicate on TSA. After 4 days of incubation, CFU were counted and expressed by the log_10_ value per spleen. The CFU data were normalized by log transformation and evaluated by ANOVA followed by Dunnett's *post hoc* test (Prism 5.0; GraphPad Software, Inc.). The experiment was repeated twice with similar results.

### Serum resistance

A serum resistance test was performed as previously described [38]. Briefly, bacteria were grown in appropriate media at 37°C until early-logarithmic phase (OD at 600 nm of 0.3–0.5). Then the bacteria were washed with PBS-MgCl_2_ 5 mM and resuspended in the same buffer. A dilution was made with the same buffer to obtain a suspension of an OD of 0.01 at 600 nm. Cells were incubated at 37°C in porcine serum (8% for *E. coli* strains and 50% for *B. suis* strains). After 0 and 60 (for *E. coli*) or 90 minutes (for *B. suis*) serial dilutions were plated to determine the CFU, and the percentage of surviving bacteria, relative to the control strains (100%) was calculated. As a control, complement was inactivated by incubating the serum at 56°C for 30 minutes [39]. The killing experiment was repeated in triplicate three times with similar results.

### Evaluation of antibody response by Enzyme-Linked Immunobsorbent assay (ELISA)

96 well ELISA microplates (Greiner bio-one REF655061) were covered with inclusion body fractions of *E. coli* pBBR*btaE* [20], *E. coli* pBBR*btaF* or *E. coli* pBBR1MCS (as control). These preparations were diluted in sodium carbonate buffer 0.06 M pH 9 to a final concentration of 10 µg/ml, and incubated for two hours at room temperature, with gentle agitation. Free sites were blocked by incubating overnight at 37°C with PBS-casein 0.1%. After washing with 0.05% PBS-Tween 20 (PBS-T), serum from healthy or sick pigs donors (1∶1000) was added and incubated overnight at 4°C. After washing with PBS-T, Protein A/G-Calf Intestinal Alkaline Phosphatase conjugated (Thermo Scientific, 1∶5000) was added. The reaction was developed with 4-nitrophenyl phosphate (Sigma). The resulting colour was read at 405 nm in a Microplate Reader (DTX 880 multimode detector, Beckman Coulter). Values from control wells were subtracted from the experimental samples prior to analysis to control for non-specific binding. The reported data represent the results of sera from 14 sick and 14 healthy pigs assayed each one in triplicate. Data were analysed by Students test. Sera were kindly provided by Sebastián Elena, Ana María Nicola and Cristina Franco from Brucellosis Department, DILAB, Servicio Nacional de Sanidad y Calidad Agroalimentaria (SENASA).

## Results

### BtaF, a new member of the TA family from *B. suis*

*In silico* analysis of putative adhesins of *B. suis* [20] showed that, in addition to BtaE, another predicted protein corresponding to BR_1846 displays the characteristic precursor domains of the TA family [40], [41], [42], [43]. Indeed, BR_1846 harbours a putative N-terminal signal peptide with a predicted signal cleavage site between the residues 29 and 30 (alanine and proline), a homologue YadA-like C-terminal region [30], and an extension of 160–170 amino acids between both regions (Fig. 1), which corresponds to the functional passenger domain. Therefore, we named BR_1846 as BtaF (for *Brucella* trimeric autotransporter). Analysis of the BtaF passenger domain did not show the presence of conserved adhesion motifs or any other domain (Fig. 1). In contrast, we have previously shown that the passenger domain of BtaE comprises several repetitions of two motifs associated with adhesion (Fig. 1) [20]. Proteins of the TA family have been found to display similar architectural structures, which consist of a globular head, a connector, a stalk (corresponding to the passenger domain) and an anchor domain (corresponding to the C-terminal region), similar to a lollipop-shaped structure [44]. Using the TA Domain Annotation (daTAA) server [30] we analysed the BtaF passenger domain. This analysis only showed the presence of several regions associated with the stalk (Fig. 1). Instead, no regions corresponding to the head or the connector were predicted. Unlike BtaF, we have previously shown that the BtaE passenger domain harbours several domains associated with the globular head, the connector and the stalk (Fig. 1) [20].

Interestingly, secondary structure prediction of the BtaF passenger domain using Metaserver [45] suggested structural similarity to an adhesion domain (PDB: 2qih_A) of the UspA1 adhesin, a TA from *Moraxella catarrhalis* [46].

BtaF orthologues were identified in the genomes of all the species/strains of *Brucella* analysed (Table 1). Although a BtaF homologue was not annotated in the genome of *B. ovis* ATCC 25840, an open reading frame encoding a full length protein was identified. All the homologues display the characteristic (and essential) functional domains of TAs. Interestingly, we found significant differences between the different orthologues. The protein length varies from 155 (for *B. melitensis* BMEI0205) to 437 amino acids (for *B. microti*) (Table 1). Furthermore, while the passenger domains of several BtaF homologues (including that one of the *B. suis* 1330 protein) do not display head domains, other homologues display one or two of these domains. The percentage of similarity of the passenger domains of the different orthologues shared with that one of BtaF of *B. suis* 1330 varies from 67% (for BAB1_1854 from *B. abortus* 2308) to 96% (for *B. pinnipedialis*). To note, while the passenger domains of the BtaF homologues of *B. suis* strains 1330, VBI22 and 92/29 were highly conserved, the passenger domain of the protein of *B. suis* bv. 3 str. 686 differs in the presence of two head structural domains and was only 73% similar to BtaF of 1330. Therefore, BtaF orthologous proteins show variations not only among distinct *Brucella* species, but also between different strains of the same species.

**Table 1:** Comparison of BtaF orthologues from different species and strains of *Brucella*.

Strain	Locus	Length (aa)	daTAA annotation^a^				Identity/Similarity of passenger domain^b^
			Head	Stalk	Connector	Anchor	
*B. suis* 1330	BR_1846	278	-	1	-	1	-
*B. suis* VBI22	BSVBI22_A1842	278	-	1	-	1	100/100
*B. suis* 92/29	C062_01978	311	-	1	-	1	99/100
*B. suis bv.* 3 str. 686	EEY31924	269	2	1	-	1	70/73
*B. abortus* 2308	BAB1_1854	197	2	2	-	1	65/67
*B. abortus* s19	BAbS19_I17340	257	2	2	-	1	72/74
*B. melitensis* bv. 1 16 M	BMEI0205	155	-	1	-	1	84/85
*B. canis* ATCC 23365	BCAN_A1884	374	-	2	-	1	75/75
*B. microti*	BMI_I1862	437	1	2	-	1	85/89
*B. pinnipedialis*	BPI_I1902	311	-	2	-	1	95/96
*B. ovis* ATCC 25840	Present^c^	195	-	1	-	1	92/93

a Domains as determined by daTAA (http://toolkit.tuebingen.mpg.de/data). Numbers of domains predicted to be part of the head, stalk, connector or anchor are indicated.

b Percentage of Identity/Similarity shared with BtaF from *B. suis* 1330.

c Locus not annotated; a gene encoding a predicted full length protein homologue was identified between positions 1786495 and 1785911 (585 nucleotides) of chromosome I.

### BtaF is involved in adhesion to ECM components and abiotic surfaces

**Figure 2:**
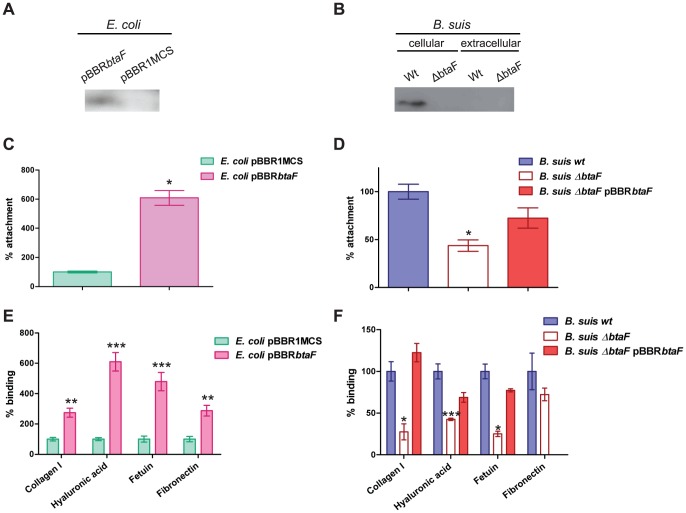
Role of BtaF in the adherence of *B. suis* to an abiotic surface and ECM components. Expression of BtaF was evaluated in *E. coli* (A) and *B. suis* (B) strains by Western blot analysis. Total cell proteins and secreted proteins were separated on a semi-native gel (10%) and transferred to PDVF membrane. Binding to the wells of PE microtiter plates (C and D) or to immobilized ligands (E and F) of *E. coli* pBBR*btaF* and *E. coli* pBBR1MCS (C and E), *B. suis* wild type, *B. suis* Δ*btaF* and *B. suis* Δ*btaF* pBBR*btaF* (D and F) was assayed. Values correspond to the percentage of attachment to the PE surface or percentage of binding to ECM components. A value of 100% was assigned to control strains. Data represent the means ± and standard deviations (SD) of the results of a representative experiment done in triplicate. Three independent experiments were performed with similar results. Data were analyzed by Student's *t* test and by one-way ANOVA followed by a Tukey *a posteriori* test. *, significantly different from control (*P*<0.05), with 95% confidence.

In order to study whether BtaF is involved in the adhesive properties of the bacterial cell surface, a heterologous strategy and a mutational approach were performed. The *btaF* gene with its own promoter was cloned into the pBBR1MCS-1 vector to generate pBBR*btaF*. This plasmid was transferred to the non-adhesive *E. coli* strain K12. Expression of BtaF in the heterologous host was analysed by Western blot. Antibodies against a fragment of the BtaF passenger domain (Fig. 1, underlined region) were generated in mouse and purified by standard methods. Since trimeric autotransporters are very difficult to denature (because the C-terminal domains of TAs form heat and SDS-resistant trimmers), we decided to solve these proteins on semi-native gel electrophoresis to favour the trimeric form. Proteins were immunodetected with anti-BtaF antibodies. A band corresponding to BtaF was detected in *E. coli* carrying pBBR*btaF* but was absent in the control strain carrying the empty vector (Fig. 2A), indicating that BtaF is expressed in the heterologous host. A knock-out mutant strain of *B. suis* was generated by a clean deletion of the *btaF* gene. Expression of BtaF in bacteria grown in TSB was evaluated by Western blot as described above. All TAs characterized so far remain associated with the cell surface. To confirm this prediction, culture supernatants and whole-cell fractions of the wild type and Δ*btaF* mutant strains were analyzed by gel electrophoresis and Western blot. A protein band recognized by the anti-BtaF antibodies was observed in the whole-cell fraction of the wild type strain, while this band was absent in the same fraction of the mutant (Fig. 2B). Instead, BtaF was not detected in the culture supernatants of the wild type or the mutant. These observations indicate that BtaF is expressed by wild type bacteria under conditions assayed and confirm that the passenger domain remains bound to the bacterial surface.

We first analysed the ability of BtaF-expressing *E. coli* cells to attach to hydrophobic surfaces in comparison with that of the control strain that carries the empty vector. To this end, the different strains were grown in a PE multiwell plate and attached bacteria were estimated by CV staining. *E. coli* pBBR*btaF* showed a six-fold increase in the attachment to PE compared with the control strain (Fig. 2C). In contrast, no differences were observed in the binding to a hydrophilic glass surface between *E. coli* pBBR*btaF* and the control strain (data not shown). In line with these observations, a significant decrease (by 56%) in the attachment to PE of the Δ*btaF* mutant compared with the wild type strain was observed (Fig. 2D). The pBBR*btaF* plasmid restored attachment to the abiotic surface confirming the role of BtaF in the formation of a biofilm *in vitro*. We have previously shown that deletion of *btaE* does not affect bacterial attachment to PE [20]. To test the possibility of overlapping functions between BtaE and BtaF, we evaluated the attachment phenotype of a double mutant Δ*btaE* Δ*btaF*. CV staining showed no significant differences between the double mutant and the Δ*btaF* single mutant (data not shown), strongly suggesting that BtaF (but not BtaE) participates in the formation of a biofilm *in vitro*.

The BmaC monomeric autotransporter mediates the binding of *B. suis* to fibronectin [19]. Recent data showed that BtaE TA contributes to the binding of *B. suis* to hyaluronic acid [20]. To test the possibility that BtaF is also involved in the binding to components of the ECM, we analysed the binding of *E. coli* pBBR*btaF* to different ECM components. We found that this strain was able to bind more efficiently to several substrates such as, collagen, hyaluronic acid, fetuin (a sialic acid rich protein) and fibronectin, compared with the control strain (Fig. 2E). In agreement with these observations, the *B. suis* Δ*btaF* mutant showed a significant decrease in the ability to bind collagen, hyaluronic acid and fetuin compared with the wild type strain. Accordingly, wild type binding levels were restored by the pBBR*btaF* plasmid (Fig. 2F). The absence of a defective adhesion phenotype of Δ*btaF* towards fibronectin may be due to overlapping functions between BtaF and other adhesins such as BmaC [19].

The phenotype of the Δ*btaF* mutant may be due to a pleiotropic effect on the bacterial surface. To evaluate this possibility we assayed the resistance of the mutant to several toxic compounds, which can be related to cell envelope composition. We found that the Δ*btaF* strain shows the same level of resistance to either 0.1% Triton X-100, 0.1% SDS, 200 µg/ml EDTA, 0.1% sodium deoxycholate or different concentrations of the cationic peptide Polymyxin B compared with the wild type strain (not shown); thus, the possibility that the *btaF* deletion causes a pleiotropic effect seems unlikely.

Taken together, these results indicate that BtaF is involved in the adherence to a variety of surfaces including a hydrophobic abiotic surface, suggesting that a rather nonspecific adhesiveness is conferred by BtaF. The possibility exists that a function of BtaF in cell-to-cell interactions may account for these adhesive properties. Since autoagglutination involves adhesion between bacteria, the settlings kinetics of bacterial aggregates was studied in liquid cultures of *E. coli* pBBR*btaF* and the control strain. Yet, no differences in the settlings kinetics between both strains were observed. Furthermore, *B. suis* Δ*btaF* showed no significant differences in the autoagglutination kinetics compared with the parental strain. Similarly, the *B. suis* Δ*btaE* Δ*btaF* double mutant was not affected in autoaggregation (not shown). Therefore, it seems that BtaF influences attachment to various immobilized ligands and to hydrophobic surfaces mostly through direct interaction with these surfaces.

### Role of BtaF in adhesion to host cells

**Figure 3:**
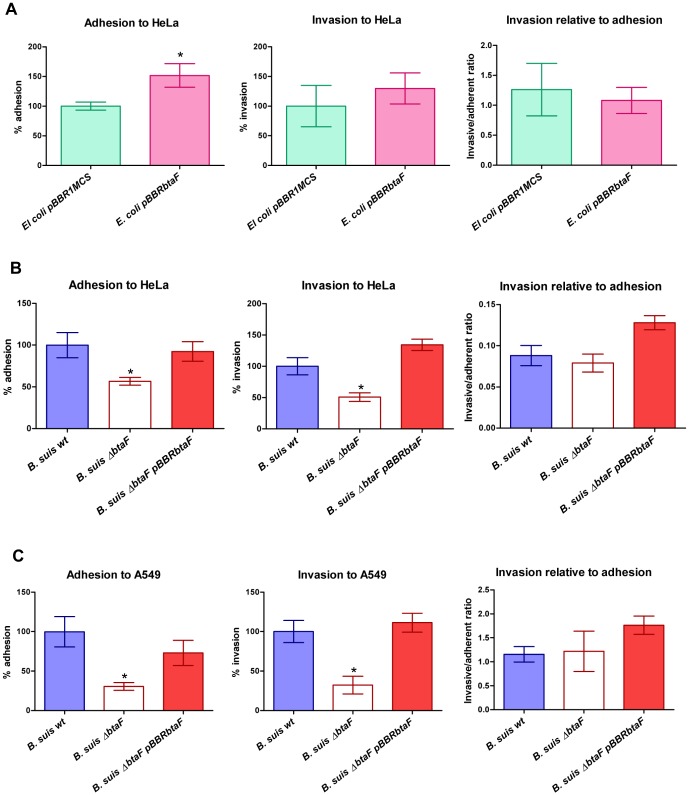
Adhesion and invasion to HeLa and A549 cells. Total numbers of adherent and intracellular (invasive) bacteria were determined, and the invasive/adherent cell ratio was calculated. HeLa cells were infected with *E. coli* pBBR*btaF* and *E. coli* pBBR1MCS (A). HeLa (B) and A549 (C) cells were infected with *B. suis* wild type, *B. suis* Δ*btaF* and *B. suis* Δ*btaF* pBBR*btaF*. Values are expressed relative to the control strains (*E. coli* pBBR1MCS or the wild type strain of *B. suis*), defined as 100%. Values represent the means ±SD of the results of a representative experiment of three independent assays done in triplicate. Data were analyzed by Student's t test and by one-way ANOVA followed by a Tukey *a posteriori* test. *, significantly different from control (P<0.05).

To evaluate whether BtaF contributes to adhesion to host cells, the ability of *E. coli* expressing *btaF* to adhere and invade HeLa cells was compared to that of the control strain. The number of adherent bacteria was calculated as the difference between total cell-associated and intracellular bacteria. The *E. coli* strain expressing BtaF showed a 51% increase in the number of adherent bacteria (2,65×10^5^ CFU/ml) compared with the number of adherent bacteria (1.75×10^5^ CFU/ml) of the control strain (considered as 100%), but no significant differences were found in invasion or in the invasive/adherent ratio (Fig. 3A).

We next evaluated the effect of *btaF* deletion in the adhesion and invasion of *B. suis* to HeLa cells. This cell line has been extensive used as non-professional phagocytic cell model to study *Brucella* internalization. In line with observations made by the heterologous approach, the Δ*btaF* mutant of *B. suis* showed a 45% reduction in the adherent bacteria (3.51×10^6^ CFU/ml) compared with the wild type strain (6.20×10^6^ CFU/ml), while adhesive levels were restored in the complemented strain (Fig. 3B). Although the mutant showed a 49% reduction in invasion compared with the wild type, no significant differences in the invasive/adhesive ratio were found between the mutant and the parental strain (Fig. 3B), indicating that BtaF is involved in the initial attachment of *B. suis* to HeLa cells rather than in cell internalization. To confirm these findings, adhesion to human lung alveolar epithelial A549 cells was assessed. The A549 cell line has been successfully used to study initial stages of *Brucella* infection [19]; [20]. *B. suis* Δ*btaF* showed a 69% reduction in the number of adherent bacteria (2.15×10^7^ CFU/ml) compared with the wild type strain (5.69×10^7^ CFU/ml) (Fig. 3C). Again, the reduction in invasion was a consequence of a decrease adhesion since no differences were observed in the invasive/adhesive ratio (Fig. 3C). As expected, the complemented strain regained the wild type adhesion phenotype.

We have recently shown that deletion of *btaE* reduces adhesion of *B. suis* to HeLa and A549 cells to levels similar to those observed in this work for the Δ*btaF* mutant [20]. To evaluate possible redundant functions of the TAs in the adhesion of *B. suis* to these cells, the Δ*btaE* Δ*btaF* double mutant was also assayed. Surprisingly, the double mutant showed similar levels of attachment to both HeLa and A549 cells than the single mutants (data not shown). A possible interpretation for this result might be that BtaE and BtaF are implicated in the same adhesion mechanism to these cell types.

Since infection of macrophages is crucial in the spread and persistence of *Brucella* spp. in the infected mammal [5], [6], we also evaluated whether BtaE and/or BtaF contribute to adhesion or invasion to professional phagocytes using as model the murine macrophage cell line J774. We have previously shown that a mutant defective in the BmaC adhesin is impaired in adhesion to epithelial cells but not to macrophages [19]. Similarly, no significant differences in adhesion, invasion or invasive/adhesive ratio were found between the wild type strain, the Δ*btaF* and Δ*btaE* single mutants or the double mutant (data not shown).

Finally, to test the possibility that the *ΔbtaF* mutant is affected in intracellular survival, a classical intracellular proliferation assay was carried out using macrophages and HeLa cells. In both cell models, the wild type and the mutant strain showed the same kinetics of intracellular replication (data not shown). Thus, similar to BmaC [19] and BtaE [20], the absence of BtaF does not affect later stages of cellular infection.

### Surface-exposed unipolar localization of BtaF

**Figure 4:**
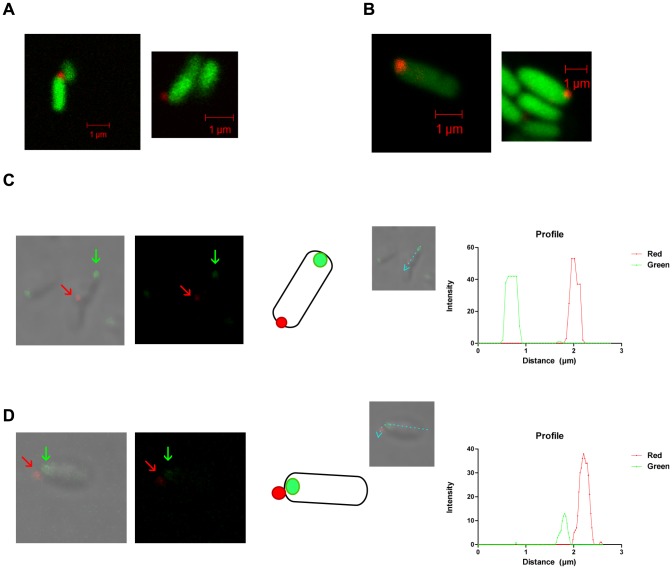
BtaF localization. Detection of BtaF (in red) on the *B. suis* wild type surface (A) and on the *E. coli* pBBR*btaF* surface (B) by immunofluorescence of GFP-tagged bacteria. Cultures of GFP-labeled strains were fixed without permeabilization, incubated with anti-BtaF antibodies, and then probed with a CY3-conjugated donkey anti-mouse antibody. Samples were observed with a Plan-Aprochromat 100×/1.4 oil DIC objective on a Zeiss LSM 5 Pascal confocal microscope. Immunofluorescence microscopy using anti-BtaF antibodies of fixed cultures of *B. suis* strains expressing Pdhs-eGFP (C) and AidB-YFP (D) as old and new pole markers, respectively. Samples were observed with a confocal LSM 510 Meta microscope, using a Plan-Aprochromat 60×/1.4 oil DIC objective. Representative images are shown. BtaF is indicated with red arrows, and pole markers with green arrows. A schematic representation is presented. Lines indicate how the intensity profile (expressed in arbitrary units) was constructed.

Both, the monomeric BmaC autotransporter and the BtaE TA display a surface unipolar localization. Interestingly, both proteins were always detected at the new pole [20]. To assess the surface presentation and localization of BtaF, late-exponential phase cultures of *B. suis* wild type or the Δ*btaF* mutant labelled with green fluorescent protein (GFP) were refreshed for 1 h in TSB medium and then fixed without permeabilization. By immunofluorescence and confocal microscopy we found that BtaF was detected in 3–4% of wild type bacteria examined, and in all cases (n = 50), the red signal corresponding to BtaF exhibited unipolar localization. In some cases the signal showed sub-polar localization (Fig. 4A). As expected, no red signal was observed on the surface of the Δ*btaF* mutant but the unipolar signal was restored in the complemented strain harbouring pBBR*btaF* plasmid (data not shown). The BtaF presentation and localization was also evaluated in *E. coli* pBBR*btaF*. The heterologous host carrying BtaF showed a red signal in 4% of the population, and in all cells it was located at one pole (Fig. 4B). No signal was observed in the control strain (data not shown).

In order to determine whether BtaF is localized at a specific pole, we used PdhS and AidB proteins as markers of the old and new poles, respectively [21], [36]. We observed that every time BtaF (in red) was detected in bacteria carrying PdhS-eGFP protein (green), it was localized at the pole opposite to PdhS (27 occurrences) (Fig. 4C), and it was never observed that BtaF and PdhS co-localized. Instead, BtaF was at the same pole as AidB-YFP (green) (40 occurrences) (Fig. 4D) or in cells with diffuse marker, but never at the opposite pole. These results suggest that similar to BmaC and BtaE, BtaF is localized at the new pole generated after cell division. Thus, it appears that these adhesins are expressed at one particular pole.

### The BtaF adhesin contributes to serum resistance

**Figure 5:**
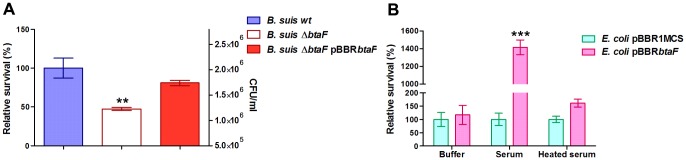
BtaF is involved in resistance to porcine serum. Early logarithmic phase cultures were used to evaluate resistance to porcine serum of *B. suis* (A) and *E. coli* (B) strains. Survival after one hour of incubation in serum with complement activity was evaluated and then expressed as survival percentage (%) relative to wild type *B. suis* 1330 or *E. coli* pBBR1MCS-1, defined as 100%. Total CFU/ml are also indicated in the right Y-axis for *Brucella* survival (A). *E. coli* survival was also assessed in buffer and heated serum (B). Values represent the means ±SD of the results of an experiment representative of three independent assays done in triplicate. Data were analyzed by Student's t test and one-way ANOVA followed by a Tukey *a posteriori* test. *, significantly different from control (P<0.05).

The activation of the complement on the bacterial surface plays a crucial role in the immune response against pathogens. However, *Brucella* is rather inefficient to activate the complement [7], [8]. It was proposed that the particular structure of *Brucella* lipopolysaccharide (LPS) reduces deposition of complement activating components [7], [8], [9], [47]. It has also been suggested that other molecules may contribute to resistance to the bactericidal activity of the complement [47]. As mentioned earlier, we found that some portions of BtaF show structural similarity with the UspA1 TA from *M. catarrhalis*. This pathogen interferes with the classical complement activation pathway by binding the complement inhibitor C4BP to UspA1 and UspA2 [48], [49]. Thus, we wondered whether BtaF might be involved in resistance to the complement present in porcine serum. To test this possibility, the effect of *btaF* deletion on *Brucella* survival in the presence of 50% porcine serum was analysed. Interestingly, the *btaF* mutant showed a significant reduction (by about 53%) in survival compared with the wild type strain, while the survival phenotype was restored by the complementing plasmid (Fig. 5A). We have reported that deletion of *btaE* does not affect serum resistance [20]. To rule out overlapping effects, survival of the *btaE btaF* double mutant was evaluated. Still, the double mutant exhibited a percentage of survival in porcine serum similar to that of the single *btaF* mutant (data not shown) suggesting that within the TAs, only BtaF is involved in serum resistance.

To further investigate whether BtaF is directly involved in serum resistance, survival in 8% porcine serum of *E. coli* strain expressing BtaF was analysed. Since it is possible that porcine serum contains antibodies against *E. coli* surface components, serum killing of *E. coli* may proceed through antibody-dependent or antibody-independent complement pathways. Interestingly, *E. coli* pBBR*btaF* showed more than ten-fold increase in the survival percentage compared with the control strain (Fig. 5B). Instead, both strains showed similar levels of survival in heat-inactivated porcine serum (Fig. 5B), suggesting that BtaF is involved in the resistance to complement-mediated serum killing.

### Antibody response to the Bta adhesins

**Figure 6:**
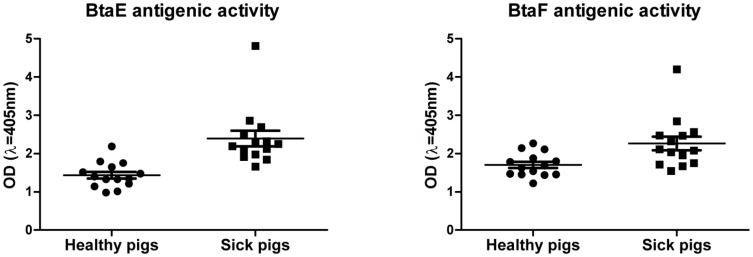
*In vivo* expression of BtaE and BtaF in pigs. Extracts from *E. coli* pBBR*btaE*, *E. coli* pBBR*btaF* and the control *E. coli* pBBR1MCS were used to perform indirect ELISA using sera from healthy and sick pigs infected with *Brucella*. The values correspond to the means ±SD of the results of a representative experiment of three independent assays done in duplicate. Data were analyzed by Student's t test. Values between healthy and sick pigs were significantly different (P<0.001 for BtaE and P<0.05 for BtaF)

To evaluate whether the TAs of *B. suis* are expressed *in vivo* during the course of infection and if they are capable to induce an immune response in the natural host, an indirect ELISA assay using sera from 14 healthy and 14 sick donor pigs was performed. We probed the different sera by ELISA with cell extracts obtained from *E. coli* pBBR1MCS, *E. coli* pBBR*btaE* or *E. coli* pBBR*btaF*. The optical density values corresponding to the control (*E. coli* pBBR1MCS) extract was subtracted from the values corresponding to the *E. coli* pBBR*btaE* or *E. coli* pBBR*btaF* extracts. The average signal (measured as OD_405nm_) obtained for sera from sick animals was significantly higher than that from healthy donors for both BtaE and BtaF extracts (Fig. 6). These results suggest that the TAs are expressed *in vivo* in the natural host and that in sick animals these adhesins induce some antibody response.

### BtaF is required for full virulence of *B. suis* in mice

**Figure 7:**
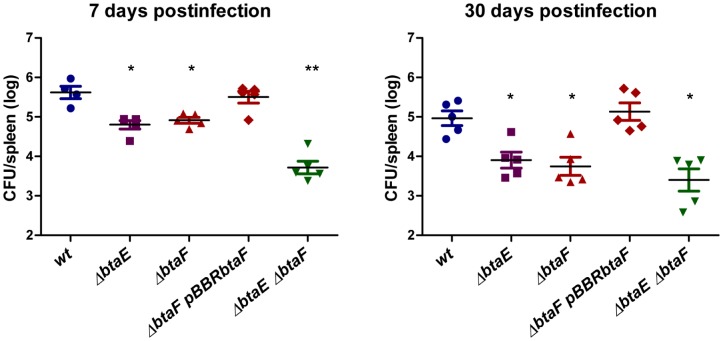
Both, BtaE and BtaF, are required for full virulence of *B. suis* in mice. BALB/c mice were inoculated with the *B. suis* strains by intragastric delivery, sacrificed at 7 and 30 days p.i., and spleens were removed. Dilutions of spleen homogenates were plated and CFU were counted and expressed as the log_10_ value per spleen. The CFU data were normalized by log transformation and evaluated by one-way ANOVA followed by Tukey's *a posteriori* test. The experiment was repeated twice with similar results. *, significantly different from the wild type (P<0.05); ** Significantly different from wild type and simple mutants (P<0.05).

To evaluate the impact of BtaF in the virulence of *B. suis*, we used the mouse infection model. Groups of five mice were infected by intragastric inoculation [50] and splenic infection was evaluated at 7 and 30 days p.i., which represent early and stabilized infection times [51]. We have previously shown that the Δ*btaE* mutant exhibits an attenuated phenotype [20]. To assess the overall impact of TA adhesins in the *B. suis* virulence, the Δ*btaE* Δ*btaF* double mutant and the Δ*btaE* single mutant were also included in the assay. At 7 days p.i. the splenic infection of *B. suis* Δ*btaF* was significantly reduced (by 0.71 log) compared with the wild type strain, while the complemented strain recovered bacterial counts (Fig. 7). A similar reduction in splenic infection was observed for *B. suis* Δ*btaE* (Fig. 7) [20]. Interestingly, at 7 days p.i. the Δ*btaE* Δ*btaF* double mutant showed a further reduction (by 1.9 log) in splenic infection compared with the wild type (Fig. 7). At 30 days p.i. the splenic infection of *B. suis* Δ*btaE* and *B. suis* Δ*btaF* single mutants was reduced by 1.06 log and 1.22 log, respectively, while the double mutant was reduced by 1.56 log, compared with the wild type strain. Thus, at 30 days p.i., infection with the strain lacking both *btaE* and *btaF* resulted in fewer CFU than the individual Δ*btaE* and Δ*btaF* mutant strains. To note, this difference was not statistically significant, probably due to dispersion of Δ*btaE* Δ*btaF* double mutant data (Fig. 7).

These results show that BtaF is required for full virulence of *B. suis* in mice infected through the oral route. The observation that at 7 days p.i., fewer CFU were recovered from the spleen of mice infected with the double mutant compared with the single mutants suggests that both, the BtaE and BtaF adhesins, are required for a full infective phenotype, especially at early stages of infection, and that some of the BtaE and BtaF roles do not overlap.

## Discussion

Initial adhesion to host components has proved to represent an important event in the pathogenesis of several pathogens. Evidence gained in recent years show that binding to the host cell surface is also relevant for *Brucella* infection [15], [16], [19], [20], [52], [53], [54]. In this work, we demonstrated that in addition to the BmaC monomeric autotransporter and the BtaE TA, a new member of the TA family, named BtaF, contributes to the adhesive features of *B. suis*. While BtaE is a relatively large protein of 740 amino acid residues and displays several predicted adhesion motifs, BtaF is a rather small adhesin of only 278 amino acid residues with no recognizable or characterized conserved domains other than the C-terminal anchor domain. Besides, although BtaF of *B. suis* only contains modules predicted to be part of the “neck/stalk” (but no one characteristic of the “head”), it is clear that BtaF is able to function as an adhesin. In line with these observations, some adhesive functions of the BadA TA from *Bartonella henselae* were independent of the presence of the head [55]. In addition, we have not found any significant protein sequence similarity between the passenger domains of BtaF and any other TA protein. Nevertheless, *in silico* analysis using Metaserver [56] suggests significant structural homology of the 57–201 region of BtaF with the structure of the 527–665 region of UspA1 (2qih_A), a TA from *M. catarrhalis*. It was proposed that this UspA1 fragment mediates the binding of *M. catarrhalis* to the human carcinoembryonic antigen-related cell adhesion molecule 1 (CEACAM1) receptor [46]. It will be interesting to evaluate whether *B. suis* interacts with the CEACAM1 molecule and if this is so, whether BtaF is involved in this interaction.

Our previous evidences showed that BmaC mediates the binding of *B. suis* to fibronectin and in lower degree to collagen, and that BtaE is involved in the binding to hyaluronic acid [19], [20]. In contrast to these adhesins, BtaF confers affinity to several surfaces, including ECM components and a hydrophobic abiotic surface. In effect, BtaF expression in a “non-adherent” *E. coli* strain was found to greatly increase the binding of recombinant bacteria to all these surfaces. Analysis of the adhesion phenotypes of the knockout *btaF* mutant confirmed these observations. Thus, BtaF confers a promiscuous adherence to several surfaces. A similar observation was made for the BadA autotransporter from the closely related *Bartonella henselae*. In this case, it was proposed that the higher is the number of neck motifs within the passenger domain, the greater is the affinity of the BadA homologue/variant to several ligands [55].

The ability to develop microcolonies or a biofilm on host surfaces could influence bacterial invasion to host cells. We have previously shown that neither of the BmaC and BtaE adhesins are involved in the formation of a biofilm *in vitro* or in the ability of *B. suis* to autoaggregate [19], [20]. We found that heterologous expression of BtaF in *E. coli* greatly increased the formation of a biofilm biomass on a hydrophobic abiotic surface. Accordingly, the *B. suis btaF* mutant was impaired in biofilm formation. The development of a biofilm depends on both, the initial attachment to the surface, and cell-to-cell interactions. However, our observations suggest that BtaF is not involved in cell-to-cell interactions. We can conclude that BtaF participates in bacterial attachment to several surfaces.

We showed that at least three adhesins of *B. suis* (BmaC, BtaE and BtaF) are required to observe a wild type adhesion phenotype to epithelial cells. While inactivation of *bmaC* reduces the adherence of *B. suis* to host cells by 2 log, deletion of either *btaE* or *btaF* consistently diminishes the binding of *B. suis* by about 50–60%. This suggests a more critical role of BmaC in the adherence to cultured cells. Intriguingly, the double mutant *btaE btaF* behaved *in vitro* similarly to the single mutants. Thus, we can hypothesize that BtaE and BtaF TAs might be part of the same “adhesion pathway” to cultured epithelial cells, each one fulfilling different and complementary functions. Further studies are necessary to understand the interplay between these two adhesins in the binding to host cells.

*Brucella* prevents detection by complement [7], [57], [58]. It was proposed that the distinctive structure of *Brucella* LPS reduces deposition of complement components [8], [9], [47]. Interestingly, it was proposed that other surface factors mediate the varied sensitivity of *Brucella* species to the bactericidal action of serum. In effect, rough strains of *B. melitensis* were shown to bind less complement than rough *B. abortus* strains. In other words, it seems that the presence of a particular exposed LPS on the bacterial surface cannot be the only factor causing the inhibition of binding of complement components observed in the more virulent organisms [47]. Our observations suggest that BtaF might be an additional factor involved in protecting *B. suis* cells from killing by the complement. In contrast, BmaC and BtaE were found not to participate in resistance to complement [19], [20]. Although previous evidence suggests that the alternative pathway is not activated by smooth or rough strains of *B. abortus* and *B. melitensis* strains, it was proposed that the classical and the lectin pathways mediate complement deposition and complement-mediated killing of *Brucella*. It was suggested that both, mannose binding lectin (MBL) and C1q (the first subcomponent of the classical complement pathway), initiate antibody-independent complement activation and killing of O-antigen-defective strains [47]. Therefore, BtaF may be involved in the inhibition of this activation mechanism. Another possibility is that *B. suis*, in contrast to *B. abortus* and *B. melitensis*, is able to activate to some extent the complement through the alternative pathway and therefore, BtaF somehow inhibits this pathway. Several surface proteins, including adhesins of the TA family were shown to be involved in serum resistance of other pathogens. *Bordetella pertusis* expresses several factors during the virulence phase that interact with complement components or its regulators, either directly or indirectly and thereby mediating resistance to complement [59]; some of them are the BrkA [60] and the Vag8 [59] autotransporters. As mentioned earlier, the UspA2 TA from *Moraxella catharralis* was found to be involved in serum resistance [61]. Additional experiments will be carried out to understand the underlying molecular mechanism involved in BtaF-mediated serum resistance.

Several evidences demonstrate that polarity is an important feature of *Brucella* and other α-proteobacteria [20], [21], [23], [36]. In fact, it was observed that individual *Brucella* interacts with the host cell surface through one of its poles [19], [62], and we determined that BmaC and BtaE are localized to the new pole generated after cell division [20]. Similar to BtaE and BmaC, under the conditions assayed, *Brucella* expressed BtaF in a low proportion of cells, and in all cases it was localized at the new pole. Evidence reported here for BtaF reinforces the hypothesis that the new pole generated after an asymmetric division [23] is differentiated for adhesion to surfaces [20]. As discussed in a previous publication, it is possible that the low proportion of cells that show a detectable fluorescent foci corresponding to the adhesin is due to one or the combination of several reasons, such as, low expression *in vitro* of *bma* or *bta* genes, and the adhesin is only expressed in a subpopulation of bacteria depending on the bacterial cell cycle stage [21], [23]. It is interesting to speculate that this subpopulation might bind more efficiently to host cells. Curiously, it was proposed that other α-proteobacteria, such as *Caulobacter crescentus* and *Agrobacterium tumefaciens* display adhesive structures on the opposite pole, i.e., the old pole generated after an asymmetric division [23], [63], [64]. As we previously discussed, it is possible that this difference might be related to the sessile mode of life of these bacteria, in comparison with the transient adhesion of *Brucella* to the host cell surface [20].

Deletion of *btaF* reduced by about 1 log the CFU recovered from spleen at early stages of mice infected by intragastric inoculation. This attenuation was similar to that observed for the *btaE* mutant [20]. Interestingly, at 7 days p.i., the *btaE btaF* double mutant established a splenic infection that was significantly reduced compared with the single mutants. Thus, although the double mutant behaved similarly to the single mutants in cultured cells, absence of both proteins results in a more severe phenotype *in vivo* compared with the attenuation observed for the single mutants. A possible interpretation for this result is that the phenotype of the double mutant *in vivo* is a consequence of a defect in more than one function of BtaF in addition to those of BtaE. As mentioned earlier, it is possible that some of the roles might be shared or complementary between BtaE and BtaF, while others could be exclusive of BtaF or BtaE, for example the role of BtaF in serum resistance.

In an effort to evaluate whether the two TAs of *B. suis* are expressed *in vivo* in the natural host, the presence of antibodies anti-BtaE and anti-BtaF in healthy and sick pigs infected with *B. suis* was evaluated by an indirect ELISA assay. This analysis suggests that both TAs are expressed *in vivo* in swine, which reinforces the significance of these adhesins in the infection process. Further studies are required to evaluate the immunogenicity strength of the adhesins.

It is important to mention that similarly to BmaC [19] and BtaE [20] orthologues, we also found significant variations in size and domain architecture of the passenger domains of the different BtaF orthologues, not only among different species but also between different strains of the same species (Table 1). An attractive hypothesis is that BtaF and the other adhesins are host preference markers that contribute to tissue tropism of *Brucella* spp. in their primary hosts.
